# Establishing normative flexibility values for the thoracic spine of competitive male South African golfers

**DOI:** 10.17159/2078-516X/2025/v37i1a21108

**Published:** 2025-07-15

**Authors:** BE Bloemhof, CA Volkwyn, S Ferreira

**Affiliations:** Department of Sport and Movement Studies, Faculty of Health Sciences, University of Johannesburg, South Africa

**Keywords:** biokinetics, mobility, degrees of movement, posterior thorax, golf

## Abstract

**Background:**

The golf swing involves complex, multi-joint movements that require flexibility, strength, and power. However, there is limited research on thoracic spine flexibility norms among competitive male golfers in South Africa.

**Objectives:**

This study aimed to establish thoracic spine flexibility values and compare the movements on the left and right sides.

**Methods:**

Ninety-eight male golfers aged 18 and older with no spine or hip injuries participated in this descriptive, comparative, and quantitative study. Thoracic spine movements, including flexion, extension, lateral flexion, rotation, and kyphosis, were measured using the EasyAngle.

**Results:**

Average values for thoracic kyphosis were 33.6°±9.7°, flexion 32.5°±11.7°, and extension 50.2°±16.4°. Left rotation averaged 36.6°±9.8° and right rotation 38.8°±10.5°, with a significant difference between the two sides (−2.2°±9.5°; p=0.024). No significant difference was found for lateral flexion. Thoracic flexion showed a weak positive correlation with left rotation and right lateral flexion, while thoracic extension had a strong correlation with thoracic flexion ROM. Excessive thoracic flexion predicted limited extension.

**Conclusion:**

These normative values enhance understanding of thoracic spine flexibility among South African golfers and provide a reference for biokineticists to tailor training programs to improve flexibility, reduce injury risk, and optimise performance.

The golf swing is a complex, whole-body movement requiring significant control and coordination across multiple joints owing to its wide range of motion (ROM).[1,2] Competitive golfers, who often compete in prestigious tournaments like the Sunshine Tour, Asian Tour, and European Tour, must master this sophisticated motion to maintain consistent performance.[1,2,3] While it is well understood that each body segment’s movement affects the golf club’s trajectory, there is limited information on the specific flexibility requirements and variations in movement patterns among competitive male golfers in South Africa.

Flexibility is crucial in the golf swing, as it contributes to efficient movement and optimal performance.[4] Defined as the ability to move joints through their full ROM smoothly, flexibility can be affected by factors such as age, sex, and activity levels.[5,6] Declines in flexibility due to ageing can impact key performance metrics, such as ball speed and shot distance. On the other hand, excessive flexibility may lead to joint instability and a higher risk of injury. Specifically, too much or too little thoracic spine flexibility can disrupt swing mechanics and negatively affect overall performance.[7] Thus, establishing normative flexibility values for the thoracic spine is essential for optimising performance and minimising injury risk in competitive South African golfers.

The X-factor technique, which enhances clubhead speed by increasing the separation between the upper and lower body, exemplifies how flexibility can improve power.[8] However, this increased stretch can also elevate the risk of spinal injuries.[2] Despite the importance of flexibility, South African golfers have no established normative values for thoracic spine ROM. This study seeks to address this gap by providing biokineticists and trainers with essential reference data to guide assessments, identify imbalances, and develop targeted exercise programs.

## Methods

### Study design

A quantitative, descriptive, and comparative research design was utilised. Participants were recruited during the officially organised Sunshine Tour and Big Easy Tour, with prior authorisation sought from the event organisers. Eligible participants were informed about the research, and written informed consent was obtained before participation. Participants had the right to withdraw their consent at any time without repercussion.

### Ethical approval

This study was granted ethical clearance by the Research Ethics Committee and Higher Degrees Committee of the University of Johannesburg (REC-1903-2023; HDC-01-142-2023).

### Participants

Purposive sampling was conducted to recruit potential participants for the study. Male golfers actively holding a Sunshine Tour and Big Easy card and participating in professional events between January and May 2023 were invited to participate in this study. Eligible participants were healthy, competitive male golfers over 18 years old with no history of musculoskeletal injuries or surgeries involving the hip or spine in the previous six months. Golfers who had only competed in the Big Easy Tour and were ineligible for Sunshine Tour membership during the testing period were excluded from the study. This exclusion was based on the rationale that, although these individuals are classified as professional golfers, they did not meet the study’s criteria for being considered competitive golfers at the required level. Ninety-eight participants provided consent and participated in the study (n=98).

### Testing procedures

Participants were asked to complete a Physical Activity Readiness Questionnaire (PAR-Q+) before undergoing a battery of flexibility tests for the thoracic spine using the EasyAngle (Meloq AB, Stockholm, Sweden). The EasyAngle, a digital goniometer, had greater reliability than an inclinometer, particularly in terms of the smallest detectable difference (3.19–4.09 degrees) and standard error of mean values (1.15–1.48 degrees). This device proved to be an effective measurement tool with good intra-rater reliability (ICC 0.994) and inter-rater reliability (ICC 0.997–0.998), making it a superior tool for measuring flexibility compared to both the universal goniometer and the inclinometer.[9]

### Set-up and participant positioning

Participants were seated on a plinth with their hips and knees flexed at 90 degrees and arms crossed in front of the body. A yoga block was placed vertically behind the lower back to minimise compensatory lumbar movement. For thoracic rotation assessments, a small soft ball (20 cm × 20 cm) was positioned between the knees to reduce lumbar spine contribution further. Measurements were obtained using the EasyAngle digital goniometer. All measurements were performed three times, and the mean value was recorded. Measurements were taken separately for the right and left sides, with three repetitions recorded for each movement direction.

#### Thoracic flexion measurement

To measure thoracic flexion, the EasyAngle device was first positioned vertically at the T12 level, and the device was calibrated to 0°. Next, the device was placed vertically at T1 while the participant maintained a neutral seated posture. The initial measurement was recorded. The participant was then instructed to flex their upper back forward. Afterwards, the EasyAngle was reset to 0° at T12. The device was repositioned at T1 once again, and the final measurement was recorded. Thoracic flexion was calculated by subtracting the initial measurement (Step 2) from the final measurement (Step 4): *Thoracic Flexion = Step 4 – Step 2*.

#### Thoracic extension measurement

For thoracic extension, the initial degree of thoracic kyphosis was recorded by placing the EasyAngle vertically at T12 and setting it to 0°. The device was then moved to T1 while the participant maintained a neutral seated posture, and the measurement was recorded. The participant was instructed to extend their upper back. The EasyAngle was reset to 0° at T12. Once the participant completed the movement, the device was repositioned at T1, and the final measurement was recorded. Thoracic extension was calculated by adding the recorded values from Step 2 and Step 4: *Thoracic Extension = Step 2 + Step 4*.

#### Thoracic rotation measurement

To measure thoracic rotation, the participant sat with their hips and knees at 90°, maintaining a neutral trunk position and arms crossed over a dowel to minimise shoulder involvement. The EasyAngle was positioned at T1, parallel to the floor and away from the body, and calibrated to 0°. The participant was instructed to rotate maximally to one side, while the examiner followed the motion of the scapular spine. The measurement was recorded at the point of maximal rotation. Next, the EasyAngle was moved to T12, parallel to the floor, and reset to 0°. The participant repeated the maximal rotation, and the final measurement was recorded. Thoracic rotation was calculated by subtracting the final measurement (Step 4) from the initial measurement (Step 2): *Thoracic Rotation = Step 2 – Step 4*.

#### Thoracic lateral flexion measurement

For thoracic lateral flexion, the EasyAngle was positioned vertically at T12 and calibrated to 0°. The participant was instructed to bend laterally to the maximum extent possible, and the measurement at T12 was recorded. The device was then repositioned vertically at T1 and reset to 0°. The participant repeated the lateral flexion, and the final measurement at T1 was recorded. Thoracic lateral flexion was calculated by subtracting the recorded value at T12 from the value at T1: *Thoracic Lateral Flexion = Step 4 – Step 2*.

### Statistical analysis

Descriptive statistics are presented as mean±standard deviation (SD). The Kolmogorov-Smirnov normality test was used to determine the normality of data for the participants (n=98). Paired sample t-tests were used to analyse and compare the mean flexibility scores of the thoracic spine between the left and right sides to determine if there were any statistically significant differences. A correlation analysis was conducted to identify relationships and quantify the magnitude of these relationships among the mean flexibility scores of the thoracic spine for the male participants. Statistical data analysis was performed using the Statistical Package for Social Sciences (SPSS, version 28), and statistical significance was set at the alpha value of p<0.05.

## Results

### Data distribution and normality test

The Kolmogorov-Smirnov test was conducted to assess the normality of the flexibility data for each thoracic spine movement. Most flexibility measurements, including thoracic kyphosis (p=0.200), thoracic flexion (p=0.200), thoracic extension (p=0.089), thoracic rotation left (p=0.200), thoracic lateral flexion left (p=0.200), and thoracic lateral flexion right (p=0.200), followed a normal distribution. However, the correct measurement of thoracic rotation right (p=0.019) deviated from normality, with a statistically significant result of p<0.05. All other flexibility measures were normally distributed, as indicated by p-values greater than 0.05, confirming the appropriateness of parametric tests for further analyses on these variables. A confidence level of 95% was used for this analysis.

### Descriptive statistics of thoracic spine flexibility

Table 1 summarises the descriptive statistics for thoracic spine flexibility. The measurements include thoracic extension, flexion, kyphosis, rotation (left and right), and lateral flexion (left and right). Mean values, standard deviations, and range data are provided in the table for clarity.

### Comparison of left and right-side flexibility

Table 2 compares the flexibility scores for thoracic rotation and lateral flexion between the left and right sides. A significant difference was observed between thoracic rotation left and thoracic rotation right, with a mean difference of −2.2±9.5° and a significance of p=0.024. This suggests a measurable asymmetry in thoracic rotation between the left and right sides.

However, for thoracic lateral flexion, the difference between the left and right sides was −1.2°±7.3°, with a significance of p=0.100, indicating no statistically significant difference. This finding suggests that the study population’s thoracic lateral flexion was relatively symmetrical between the left and right sides.

These results provide insight into the asymmetry in thoracic rotation among competitive male golfers, while lateral flexion remained more balanced.

### Positive correlations between flexibility scores

Significant high positive correlations among flexibility scores for participants are presented in Table 3. Notably, there is a strong positive correlation between thoracic extension and thoracic kyphosis, with a Pearson correlation coefficient of r=0.81 (p<0.001). These findings suggest that as thoracic extension increases, thoracic kyphosis also tends to increase.

Table 3 displays significant low positive correlations between various flexibility scores amongst participants.

These findings suggest that as thoracic flexion increases, thoracic rotation to the left and thoracic lateral flexion to the right tend to increase among the male participants in this study. This highlights the positive relationship between thoracic flexion and other aspects of thoracic mobility.

### Negative correlations between flexibility scores

Table 4 outlines significant low negative correlations between various flexibility scores amongst participants.

## Discussion

This study provides valuable insights into the thoracic spine flexibility of competitive male golfers in South Africa, contributing to a deeper understanding of how thoracic spine range of motion (ROM) affects golf performance. Previous research has highlighted a significant connection between hip joint mobility and lower back pain in golfers.[1,3,12,15,16] The current study highlights significant asymmetries in thoracic spine rotation between the left and right sides, correlations among different flexibility measurements, and potential implications for injury prevention and performance optimisation.

### Thoracic spine flexibility and asymmetry

The results reveal a statistically significant difference in thoracic rotation between the left and right sides, with a mean difference of −2.2°. This asymmetry aligns with existing literature that suggests slight differences in ROM between sides are common in sports that require unilateral movements, such as golf.[17] As the golf swing involves a dominant rotation pattern, this asymmetry could result from repetitive motions during practice and play. The implications of such asymmetries are crucial, as imbalances in thoracic rotation may increase golfers’ risk of injury, particularly in the spine, shoulders, and hips.[18] Biokineticists should assess golfers’ thoracic rotation during routine evaluations and design targeted interventions to correct imbalances, thereby reducing injury risk and improving performance.

### Relationship between thoracic flexion, extension, and kyphosis

The strong positive correlation between thoracic extension and kyphosis (r=0.81) highlights a relationship where increased kyphosis is associated with increased thoracic extension. This finding suggests that golfers with more pronounced thoracic curvature (kyphosis) may experience compensatory increases in extension to maintain balance and posture during the golf swing. Excessive kyphosis and its association with thoracic extension could limit trunk flexibility and negatively affect swing mechanics, potentially impacting shot accuracy and distance.[12,19] These results underscore the need for training programs that address postural control and flexibility to maintain optimal thoracic spine curvature.

Interestingly, a strong negative correlation was observed between thoracic flexion, thoracic extension, and kyphosis (r=−0.49). This finding suggests that increased thoracic flexion leads to reduced extension and a flattened thoracic curve, which may further affect spinal mobility. As excessive thoracic flexion restricts thoracic extension, golfers exhibiting excessive flexion may struggle to achieve the rotational range necessary for a powerful and fluid swing.[20,21] Biokineticists can use this information to prioritise thoracic spine mobility exercises in their training protocols to counteract the adverse effects of excessive thoracic flexion.

### Implications for performance and injury risk

Flexibility in the thoracic spine is crucial for executing an efficient and powerful golf swing. The X-factor technique, which maximises the upper and lower body separation, relies on flexibility to generate increased clubhead speed. However, the study’s findings suggest a delicate balance between adequate flexibility for performance and excessive flexibility, which may increase the risk of injury.[3,18,19] For example, excessive thoracic rotation may lead to spinal instability, and a higher degree of kyphosis could exacerbate back problems. Therefore, training programs should aim to maintain thoracic spine flexibility within an optimal range to enhance performance while minimising the risk of injury.

This study also revealed significant correlations between thoracic flexibility and lateral flexion, specifically the weak positive relationship between thoracic flexion and right-side lateral flexion (r=0.26). This correlation suggests that as golfers improve their thoracic flexion, their ability to perform lateral flexion to the right may increase, which is beneficial for maintaining balance and stability during the downswing phase.[12,19] Incorporating flexibility exercises that target both flexion and lateral flexion may further enhance golfers’ ROM and swing control.

### Practical applications for biokineticists

These thoracic spine flexibility values provide valuable benchmarks for biokineticists and trainers working with competitive golfers. Practitioners can tailor programs to correct imbalances or deficits by identifying deviations from these norms. For example, golfers with asymmetrical thoracic rotation can benefit from exercises that promote balanced movement, while those with excessive kyphosis or flexion may require posture correction and exercises that focus on thoracic extension. Regular monitoring of thoracic flexibility is crucial for detecting early dysfunctions. Fast and reliable assessments, such as those using the EasyAngle device, can guide training, and this study establishes a baseline for future research in other vital areas, including the lumbar spine and hips.

### Limitations and future research

The present study faced limitations due to a small sample size of female participants, largely influenced by a decrease in female golfers on the Ladies Sunshine Tour, the migration of South African female professionals abroad, and fewer local women’s professional events. The sample, consisting exclusively of elite female golfers competing in South Africa, was further limited by excluding individuals with recent injuries, which impacted the ability to generalise findings. Additionally, assessments were conducted during tournaments without a standardised warm-up. Future research should explore standardised warm-up protocols, weight-bearing assessments, and correlations between flexibility and performance metrics like ball speed and distance. Broader studies, including more diverse participants, could improve the understanding of flexibility norms, the relationship between range of motion and injury risk, and inform comprehensive evaluation protocols for golfers globally.

## Conclusion

This study establishes normative values for thoracic spine flexibility among competitive male golfers in South Africa, providing essential data that can be used to assess thoracic mobility, identify asymmetries, and design targeted interventions. These findings have practical applications for improving golf performance and reducing the risk of injury. Future research should extend this analysis to other body segments and populations to enhance our understanding of flexibility in golf performance.

## Figures and Tables

**Table 1:** Descriptive statistics of thoracic spine flexibility (n=98)

Flexibility measurement	Mean ± SD (°)	Minimum (°)	Maximum (°)	Median
Thoracic Extension	50.2±16.4	21.0	102.0	49.0
Thoracic Flexion	32.5±11.7	−28.0	59.0	32.3
Thoracic Kyphosis	33.6±9.7	3.0	73.0	33.3
Thoracic Rotation Left	36.6±9.8	10.0	61.0	37.7
Thoracic Rotation Right	38.8±10.5	−1.0	62.0	39.7
Thoracic Lateral Flexion Left	16.9±6.9	−2.0	33.0	17.0
Thoracic Lateral Flexion Right	18.2±7.7	−3.0	41.0	18.0

° Degrees

**Table 2:** Comparison of flexibility scores of the left and right sides (n=98)

Flexibility measurement	Mean ± SD (°)	p-value	95% Confidence Interval	

			Lower	Upper
Thoracic Rotation (L vs. R)	−2.2±9.5	0.024*	−4.1	−0.3
Thoracic Lateral Flexion (L vs. R)	−1.2±7.3	0.100	−2.7	0.2

* The correlation between left and right was significant at a 0.05 level.

° Degrees

**Table 3:** Positive correlations between thoracic flexibility measures (n=98)

Flexibility measurement	Correlation (95% CI)	p-value
Thoracic extension vs. Thoracic kyphosis	0.81 (0.73 to 0.87)	0.001
Thoracic flexion vs. Thoracic rotation Left	0.24 (0.04 to 0.42)	0.017
Thoracic flexion vs. Thoracic lateral flexion Right	0.26 (0.06 to 0.44)	0.010

**Table 4:** Negative correlations between thoracic and lumbar flexibility measurements (n=98)

Flexibility measurement	Correlation (95% CI)	p-value
Thoracic extension & Thoracic lateral flexion Right	−0.28 (−0.45 to −0.09)	0.006
Thoracic kyphosis & Thoracic lateral flexion Right	−0.29 (−0.46 to −0.10)	0.004
Thoracic kyphosis & Lumbar lateral flexion Left	−0.26 (−0.44 to −0.06)	0.010
Thoracic flexion & Thoracic extension	−0.49 (−0.63 to −0.32)	0.001
Thoracic flexion & Thoracic kyphosis	−0.49 (−0.63 to −0.32)	0.001

## References

[1] Alderslade V, Crous LC, Louw QA (2015). Correlation between passive and dynamic range of rotation in lead and trail hips during a golf swing. South Afr J Res Sport Phys Educ Recreat.

[2] Joyce C (2017). The most important “factor” in producing clubhead speed in golf. Hum Mov Sci.

[3] Sell TC, Tsai YS, Smoliga JM, Myers JB, Lephart SM (2007). Strength, flexibility, and balance characteristics of highly proficient golfers. J Strength Cond Res.

[4] Marshall KJ, Llewellyn TL (2017). Effects of flexibility and balance on driving distance and club head speed in collegiate golfers. Int J Exerc Sci.

[5] Lestari AB, Alim A, Sukamti ER, Hartanto A (2023). Static vs dynamic stretching: which is better for flexibility in terms of gender of badminton athletes?. Pedagogy Phys Cult Sports.

[6] Ehlert A (2020). The correlations between physical attributes and golf clubhead speed: a systematic review with quantitative analyses. Eur J Sport Sci.

[7] Joyce C (2017). An examination of the correlation amongst trunk flexibility, x-factor, and clubhead speed in skilled golfers. J Sports Sci.

[8] von Kwon YH, Han KH, Como C, Lee S, Singhal K (2013). Validity of the X-factor computation methods and relationship between the X-factor parameters and clubhead velocity in skilled golfers. Sports Biomech.

[9] Svensson M, Lind V, Löfgren Harringe M (2019). Measurement of knee joint range of motion with a digital goniometer: A reliability study. Physiother Res Int.

[10] Lindsay DM, Vandervoort AA (2014). Golf-related low back pain: a review of causative factors and prevention strategies. Asian J Sports Med.

[11] Callaway AJ, Glaws KR, Mitchell DL (2012). An analysis of peak pelvis rotation speed, gluteus maximus and medius strength in high versus low handicap golfers during the golf swing. Int J Sports Phys Ther.

[12] Cole MH, Grimshaw PN (2016). The biomechanics of the modern golf swing: implications for lower back injuries. Sports Med.

[13] Creighton A, Cheng J, Press J (2022). Upper body injuries in golfers. Curr Rev Musculoskelet Med.

[14] Mun F, Suh SW, Park HJ, Choi A (2015). Kinematic relationship between rotation of lumbar spine and hip joints during golf swing in professional golfers. Biomed Eng Online.

[15] Deckard L (2021). A targeted approach to evaluating the golfing athlete with low back pain: a resident’s case report. Int J Sports Phys Ther.

[16] Donahue PT, Szymanski D, Wilson SJ (2020). Association of anthropometrics and physical performance measures to golf-specific variables in collegiate male golfers. J Sports Med Phys Fitness.

[17] Menzer H, Gill GK, Paterson A (2015). Thoracic spine sports-related injuries. Curr Sports Med Rep.

[18] Sim T, Yoo H, Choi A, Lee KY, Choi MT, Lee S (2017). Analysis of pelvis-thorax coordination patterns of professional and amateur golfers during golf swing. J Mot Behav.

[19] Gould ZI, Oliver JL, Lloyd RS, Neil R, Bull M (2021). The golf movement screen is related to spine control and X-factor of the golf swing in low handicap golfers. J Strength Cond Res.

[20] Joyce C (2016). An examination of the correlation amongst trunk flexibility, x-factor, and clubhead speed in skilled golfers. J Sports Sci.

[21] Chu Y, Sell TC, Lephart SM (2010). The relationship between biomechanical variables and driving performance during the golf swing. J Sports Sci.

